# Development of a new loop-mediated isothermal amplification test for the sensitive, rapid, and economic detection of different genotypes of Classical swine fever virus

**DOI:** 10.3389/fcimb.2024.1372166

**Published:** 2024-04-15

**Authors:** Jose Alejandro Bohórquez, Adriana Muñoz-Aguilera, Saraswathi Lanka, Liani Coronado, Rosa Rosell, Mònica Alberch, Carol W. Maddox, Llilianne Ganges

**Affiliations:** ^1^ Veterinary Diagnostic Laboratory, College of Veterinary Medicine, University of Illinois at Urbana-Champaign, Urbana, IL, United States; ^2^ WOAH Reference Laboratory for Classical Swine Fever, IRTA-CReSA, Barcelona, Spain; ^3^ Unitat mixta d’Investigació IRTA-UAB en Sanitat Animal, Centre de Recerca en Sanitat Animal (CReSA), Universitat Autònoma de Barcelona (UAB), Bellaterra, Barcelona, Spain; ^4^ WOAH Collaborating Centre for the Research and Control of Emerging and Re-Emerging Swine Diseases in Europe (IRTA-CReSA), Barcelona, Spain; ^5^ Instituto Colombiano Agropecuario (ICA), Bogotá, DC, Colombia; ^6^ Departament d’Acció Climàtica, Alimentació i Agenda Rural, Generalitat de Catalunya, Barcelona, Spain; ^7^ Department of Pathobiology, College of Veterinary Medicine, University of Illinois at Urbana-Champaign, Urbana, IL, United States

**Keywords:** CSFV, LAMP, diagnostic, point of care, viral RNA, surveillance, RT-qPCR

## Abstract

**Background:**

Classical swine fever virus (CSFV) remains one of the most important pathogens in animal health. Pathogen detection relies on viral RNA extraction followed by RT-qPCR. Novel technologies are required to improve diagnosis at the point of care.

**Methods:**

A loop-mediated isothermal amplification (LAMP) PCR technique was developed, with primers designed considering all reported CSFV genotypes. The reaction was tested using both fluorometric and colorimetric detection, in comparison to the gold standard technique. Viral strains from three circulating CSFV genotypes were tested, as well as samples from infected animals. Other pathogens were also tested, to determine the LAMP specificity. Besides laboratory RNA extraction methods, a heating method for RNA release, readily available for adaptation to field conditions was evaluated.

**Results:**

Three primer sets were generated, with one of them showing better performance. This primer set proved capable of maintaining optimal performance at a wide range of amplification temperatures (60°C - 68°C). It was also able to detect CSFV RNA from the three genotypes tested. The assay was highly efficient in detection of samples from animals infected with field strains from two different genotypes, with multiple matrices being detected using both colorimetric and fluorometric methods. The LAMP assay was negative for all the unrelated pathogens tested, including Pestiviruses. The only doubtful result in both fluorometric and colorimetric LAMP was against the novel Pestivirus italiaense, ovine Italy Pestivirus (OVPV), which has proven to have cross-reaction with multiple CSFV diagnostic techniques. However, it is only possible to detect the OVPV in a doubtful result if the viral load is higher than 10000 viral particles.

**Conclusion:**

The results from the present study show that LAMP could be an important addition to the currently used molecular diagnostic techniques for CSFV. This technique could be used in remote locations, given that it can be adapted for successful use with minimal equipment and minimally invasive samples. The joined use of novel and traditional diagnostic techniques could prove to be a useful alternative to support the CSF control.

## 1 Introduction

As one of the most important diseases in animal health, Classical Swine Fever (CSF) continues to be a challenge in terms of disease control (Ganges et al., 2020). It is caused by the CSF virus (CSFV), a ssRNA (+) virus, a member of the *Pestivirus* genus. CSF is currently endemic in Asia, Central and South America and parts of Eastern Europe and, due to its great impact on animal health and economy, it requires mandatory notification to the WOAH (World organisation for animal health (WOAH), 2019b). Control strategies against CSF are reliant on rapid diagnosis, primarily ELISA testing for anti-CSFV antibodies, as well as molecular detection of viral RNA using real time RT-PCR (RT-qPCR) (World organisation for animal health (WOAH), 2019a). In the case of CSF, molecular diagnostic strategies are reliant on the few conserved regions, such as the 5’ untranslated region (5’-UTR), which serves as the target for techniques recommended by the WOAH and USDA (Hoffmann et al., 2005; Eberling et al., 2011; World organisation for animal health (WOAH), 2019b). Other assays that target the RNA-dependent RNA polymerase region have also been used (Díaz de Arce et al., 1998). While proving very useful, these techniques require the use of specialized equipment that is not readily available in proximity to the farms or slaughterhouses where testing is needed. In addition, the specialized equipment, and highly qualified personnel, to carry out RT-qPCR tests are also not readily available in many developing countries. This greatly increases the time needed to obtain a result, which in turn increases the response time to control the infection in affected herds.Point-of-care diagnostic tests have gained popularity lately, as a potentially useful complement to the currently used techniques (Moehling et al., 2021). Among these, Loop-mediated isothermal amplification (LAMP) PCR poses one of the more interesting alternatives, due to the availability of different detection methods. LAMP can be performed using fluorometric detection, aided by easily portable equipment, or even by colorimetric detection, observable to the naked eye (Wong et al., 2018). Recently, a new LAMP assay was developed and validated for the early and sensitive detection of African swine fever virus (ASFV) (Bohorquez et al., 2023), a virus that also severely affects the pig sector and in many countries that are also endemic for CSFV (Cabezón et al., 2017).

Considering the advantages of this diagnostic technique, our work aimed to design and validate a new LAMP assay for the fast, sensitive, specific, and economic detection of CSFV RNA in fluorometric, and colorimetric formats to be validated side by side with the CSFV reference RT-qPCR test. To this end, representative CSFV reference strains from genotypes 1, 2 and 3 and samples collected from CSFV experimentally infected pigs were used.

## 2 Materials and methods

### 2.1 Cells and viruses

The porcine kidney cell line PK-15 (ATCC-CCL-33) was used for viral production. CSFV strains were grown in Eagle’s Minimum Essential Medium (Lonza, Basel, Switzerland) supplemented with 5% fetal bovine serum (FBS), incubated for 72 hours at 37°C at 5% CO_2_, after cell culture inoculation.

The CSFV strains Alfort/187, Brescia, Baker, C-strain, Pinar del Rio (PdR), Margarita, Catalonia01, Diepholz, Italy, Paderborn, Rostock, Uelzen, Spreda and Kanagawa were used for LAMP validation. A comprehensive list of the CSFV strains used, their genotype, and origin is provided in **Table 1**. Determination of viral titers was carried out by end-point dilution, calculated following standard statistical methods (Reed and Muench, 1938). Viral replication was monitored using Immune peroxidase monolayer assay (IPMA) (Wensvoort et al., 1986).

**Table 1:** CSFV strains used for validation.

Reference Strain	Genotype	Origin
Alfort-187	1.1	EU and WOAH Reference Laboratory for CSF, Germany
Brescia	1.2	Institute of Animal Science and Health (ID-DLO) Lelistad, Netherlands.
Baker	1.2	Central Veterinary Laboratory (CVL), Weybridge, UK
Pinar del Rio	1.4	WOAH Reference Laboratory for CSF, IRTA-CReSA, Spain
Margarita	1.4	WOAH Reference Laboratory for CSF, IRTA-CReSA, Spain
C-Strain	1.1	Boehringer Ingelheim, Colombia
Catalonia 01	2.3	WOAH Reference Laboratory for CSF, IRTA-CReSA, Spain
Diepholz	2.3	EU and WOAH Reference Laboratory for CSF, Germany
Italy	2.2	WOAH Reference Laboratory for CSF, IRTA-CReSA, Spain
Paderborn	2.1	WOAH Reference Laboratory for CSF, IRTA-CReSA, Spain
Rostock	2.3	EU and WOAH Reference Laboratory for CSF, Germany
Uelzen	2.3	WOAH Reference Laboratory for CSF, IRTA-CReSA, Spain
Spreda	2.3	WOAH Reference Laboratory for CSF, IRTA-CReSA, Spain
Kanagawa	3.4	EU and WOAH Reference Laboratory for CSF, Germany

### 2.2 CSFV genome alignment and analysis

All the CSFV full length (n=187) and partial (n=243) N^pro^ sequences available in NCBI were aligned using the BioEdit sequence alignment Editor, version 7.0.5.3 (Hall, 1999). Once a consensus sequence was produced (≈ 12421nt), it was subjected to entropy analysis, aiming to find conserved regions (entropy below 0.2) longer than 15nt in the CSFV genome. This analysis showed 25 nucleotide regions that complied with these criteria, nine of which were found between nucleotides 161 and 529 of the consensus sequence. Primer and plasmid insert design were carried out based on this region (368 nt in length).

### 2.3 Plasmid design

A 600nt consensus sequence, containing the majority of conserved regions in the CSFV genome was submitted to Integrated DNA technologies Inc. (IDT, Coralville, IA, USA) for custom gene synthesis and cloning into a plasmid vector expressing a kanamycin resistance gene (pUCIDT (Kan)). The plasmid sequence was based on the sequences obtained from NCBI an analyzed in Bioedit and considering the variability of the three CSFV genotypes. It included 318nt from the 5´untranslated region (5´-UTR) followed by 282nt from the N^pro^ and was named pSLC. Heat shock was used to transfect pSLC into competent *E. coli* (NEB^®^ DH5-alpha Competent *E. coli* (High Efficiency), New England Biolabs, Ipswich, MA) for propagation. After picking and growing selected colonies, the plasmid was purified using the QIAGEN plasmid Midi Kit (Qiagen, Hilden, Germany) and Sanger sequencing was carried out to verify the insert sequence. pSLC concentration was quantified using the Qubit™4 Fluorometer system (Thermo Fisher Scientific, Waltham, MA) and the number of plasmid copies was calculated, based on the molecular weight of the plasmid and the concentration. Plasmid ten-fold dilutions were performed, between 10^6^ and 1 plasmid copies/µL. The plasmid dilutions were used as standards and positive control for the CSFV-LAMP validation.

### 2.4 CSFV-LAMP primer design

The 368 nt sequence containing the majority of conserved CSFV genome regions was entered in the LAMP designer, version 1.16 software (Premier Biosoft, Palo Alto, CA, USA), and CSFV LAMP primers were generated. After aligning each primer sequence with the consensus CSFV genome, degenerate nucleotides were manually introduced into each primer in the positions where there was <90% homology in all the analyzed sequences. Primer sets that included any “N” degeneracies (not conserved positions in which any nucleotide could be present in the field sequences) were discarded, as were those that showed >10% of degenerate nucleotides in the primer sequence. These primers were discarded to avoid instability in the primer design, due to the multiple possible primer sequence combinations. Both the non-degenerate, and the degenerate primer sequences were compared to check for major increases in complementarity due to nucleotide degeneracies. An additional primer set was generated through combination of sequences that had been recommended by the software to be good primer candidates but had not been suggested as part of the same primer set (i.e., the B1 sequence for a primer set, was adapted to be used as the B3 primer for the new primer set), (**Table 2**).

**Table 2:** Primer sequences for the CSFV-LAMP primer sets.

Primer set	Primer	Sequence
CSFV-LAMP-JB1	F3	GAGGGACTAGCCRTAGTG
	***B3***	***CAACTCCATGTGCCATGT***
	FIP(***F1c*** +F2)	***CACRTAGCATMTCGAGGTGGG**GGTGKTCTAAGTCCTGAGT*
	BIP(B1c+ ***B2***)	CCCAAGACACACCTTAACCCT ***GCRCCCTATCAGGTCGTA***
	***LoopF***	***GAACTACTGACGACTGTCCTG***
	LoopB	GTCGCYAGGGTGAAATCAYA
CSFV-LAMP-JB2	F3	GGTGGTCTAAGTCCTGAGT
	***B3***	***CAACTCCATGTGCCATGT***
	FIP(***F1c*** +F2)	***GGTTAAGGTGTGTCTTGGGCATAG**TAGTTCGACGTGAGCA*
	BIP(B1c+ ***B2***)	GGGTCGCYAGGGTGAAATCAC**TAATAGTGGGCCTCTGCA**
	***LoopF***	***CCTCGTCCACRTAGCATMTC***
	LoopB	TGATGGGRGTACGACCTGATA
CSFV-LAMP-JB3	F3	GGTGGTCTAAGTCCTGAGT
	***B3***	***GGTCCTCAACTCCCWYTGGTTT***
	FIP(***F1c*** +F2)	***GTGATTTCACCCTRGCGACCC**GAKATGCTAYGTGGACGAGG*
	BIP(B1c+ ***B2***)	CGACCTGATAGGGYGCTGC**CAACTCCATGTGCCATGT**
	***LoopF***	***GGTTAAGGTGTGTCTTGGGCAT***
	LoopB	AGGCCCACTADYAGGCTAGTAT

In bold-Italics: sequences in reverse-complementary form.

### 2.5 Nucleic acid extraction

Viral RNA was extracted from all the viral culture and infected animal samples to be analyzed by RT-qPCR and LAMP. An initial sample volume of 200 μL was used for extraction with the IndiMag Pathogen Kit (Indical, Leipzig, Germany), following the manufacturer’s instructions. For tissue samples, maceration through a 70 μm cell strainer was performed prior to nucleic acid extraction. For the CSFV RNA detection in tissues collected from experimentally infected pigs, samples were ground in sterile water; 1 g of tissue plus 9 mL of water supplemented with 2% penicillin (10,000 U/mL) and streptomycin (10,000 U/mL). Besides magnetic-based RNA extraction, samples were also subjected to heating treatment at 94°C for 10 minutes, to test a nucleic acid extraction protocol suitable for farm conditions. This method was performed on samples diluted at a 1:10 and 1:100 ratio in sterile water.

### 2.6 RT-qPCR tests

The CSFV RT-qPCR test accredited under 17025 by the International Organization for Standardization (ISO) in the CSF WOAH reference laboratory in IRTA-CReSA was used for viral RNA detection from CSFV strains and samples from CSFV experimentally infected animals (Hoffmann et al., 2005). The USDA-validated CSFV RT-qPCR assay was used for detection of the pSLC plasmid, containing a consensus partial sequence of the CSFV 5’-UTR and N^pro^ (Eberling et al., 2011). This assay is routinely used for CSFV surveillance at the veterinary diagnostic laboratory of the University of Illinois at Urbana-Champaign (VDL-UIUC). For both techniques, Cq values below 40 were considered as positive.

### 2.7 LAMP reactions

CSFV-LAMP reactions were performed as previously described (Bohorquez et al., 2023), under the following conditions: LAMP Mastermix 1X, FIP and BIP primers (0.8µM), LoopF and LoopB primers (0.4µM), F3 and B3 primers (0.2µM) and DNA template (5µL). The mastermix used for fluorometric LAMP was ISO-004-RT (OptiGene Limited, West Sussex, UK), while colorimetric detection was performed with the WarmStart^®^ Colorimetric LAMP Master Mix (New England Biolabs, Ipswitch, MA).

Fluorometric detection was carried out using the Genie^®^ II LAMP instrument (OptiGene Limited) following amplification at 65°C for 30 minutes. Samples were considered as positive if there was a detectable amplification peak, defined as a sustained increase in normalized fluorescence compared to the negative amplification control, accompanied by the corresponding annealing temperature peak, within 0.5°C of that established by the amplification of the pSLC plasmid. Colorimetric detection was based on color change observation in the mastermix after amplification for either 30 or 60 min, using either a thermocycler (GeneAmp^®^ PCR system 2700, Applied biosystems) or a water bath set at 65°C. Samples were deemed as positive if master mix coloration was bright yellow after amplification, suspect if it was pale pink and negative if it was bright pink, using the positive and negative amplification controls as guidelines for the colorimetric differentiation.

### 2.8 Thermal stability

The thermal stability of the fluorometric and colorimetric CSFV-LAMP reactions was tested in triplicates, with a temperature gradient between 60-70°C. The LAMP reactions were performed using the pSLC DNA at a concentration of 10^4^ plasmid copies/µL.

### 2.9 Determination of CSFV-LAMP operating range

To determine the analytical sensitivity for the CSFV-LAMP test, ten-fold dilutions of pSLC DNA, starting at a concentration of 10^6^ plasmid copies/µL through 1 plasmid copy/µL were employed. Fluorometric and colorimetric reactions were carried out in triplicates and the results were compared with those obtained by the USDA-validated CSFV RT-qPCR assay.

### 2.10 CSFV-LAMP analytical specificity testing

Nucleic acid from 25 different pathogens (**Supplementary Table 1**) relevant for porcine health, were used to evaluate the exclusivity of the CSFV-LAMP primers. Additionally, RNA samples from different strains of Pestiviruses were also analyzed by the CSFV-LAMP assay. These included one strains of Bovine Viral Diarrhea virus, types I and II (BVDV-I and BVDV-II, respectively), three strains (137/4, Moredun and Frijters) of Border Disease Virus (BDV) and one isolate of the recently discovered Ovine Pestivirus (OVPV), which has been found to cross-react with the RT-qPCR accredited for CSFV diagnostic (Hoffmann et al., 2005) (Wang et al., 2020b).

To assess the diagnostic inclusivity for the CSFV-LAMP test, an extensive battery of CSFV strains available at the WOAH reference laboratory for CSF at IRTA-CReSA were employed (**Table 1**). Ten-fold dilutions of viral cultures, containing representative strains from the three CSFV genotypes (and seven sub genotypes) were performed, and RNA was extracted as previously described in section 2.5. CSFV RNA was evaluated by fluorometric, and colorimetric LAMP and the results were compared to the RT-qPCR accredited for CSFV diagnostic (Hoffmann et al., 2005).

### 2.11 CSFV-LAMP testing in plasmid DNA-spiked samples

The ability of the CSFV-LAMP assay to detect its target was evaluated using four different matrices: sera, tonsil swabs, rectal swabs and oral fluids. Six samples for each matrix were randomly chosen from the surveillance samples at the VDL-UIUC and, following dilution at a 1:10 ratio in water, were spiked with pSLC DNA at a concentration of 5 x10^2^ plasmid copies/uL. The samples were subjected to heating treatment at 100°C for 15 minutes to mimic the minimal-equipment nucleic acid extraction procedure used with the viral isolates. To discard any influence of the operator in the LAMP results, the reactions were equally and randomly divided between two operators.

### 2.12 CSFV-LAMP validation in samples from CSFV experimentally infected pigs

Samples obtained from animals experimentally infected with either CSFV genotype 1 (36 samples) or 2 (49 samples) were randomly selected and evaluated in duplicates using the CSFV LAMP assay and the RT-qPCR (Hoffmann et al., 2005). Sample matrices included: nasal swab, rectal swab, mesenteric lymph node, tonsil, spleen, and sera. For genotype 1, samples obtained from a previously performed experimental infection in domestic pigs were tested (Lamothe-Reyes et al., 2021). Briefly, 9-week-old pigs were infected with 10^5^ TCID_50_ of CSFV Margarita strain by intramuscular injection in the right neck. Serum and swab sample were collected on the day of infection and at 6 days post infection (dpi), while organ samples were obtained at the end of the trial one day later. The protocol was approved by the Ethical Committee of the Generalitat de Catalonia, Spain under the animal experimentation project number 10908.

In the case of CSFV genotype 2, samples corresponded with a previously performed experimental infection in domestic pigs, using the CSFV Catalonia 01 strain (Bohórquez et al., 2019). The infection was carried out in 3-week-old pigs, inoculated intranasally with 2.5*10^4^ TCID_50_ of virus. Serum and swab samples were taken on the day of infection and at 7 and 14 dpi and organ samples were collected at the end of the trial. The experiment was approved by the Ethics Committee for Animal Experiments of the Autonomous University of Barcelona (UAB) under number 8642, according to existing national and European regulations.

## 3 Results

### 3.1 Design and selection of CSFV-LAMP candidates

Using the LAMP designer version 1.16 software, six candidate primer set were generated. The regions selected by the software for primer design were found to overlap between multiple primer set candidates (i.e. primer sets 2 and 3 had the same sequences as primer set 1, with 2 extra nucleotides upstream or downstream in the F1 sequence), (**Table 2**). Considering this, as well as the conservation of the primer regions across all the CSFV strain genomes analyzed, two primer sets were synthesized and selected for testing (named JB1 and JB2). An additional primer set (JB3) was manually created by selecting conserved regions that had been suggested by the software as parts of different primer sets. The candidates were initially tested by fluorometric LAMP detection, with primer sequences detailed in **Table 2**.

**Figure 1:**
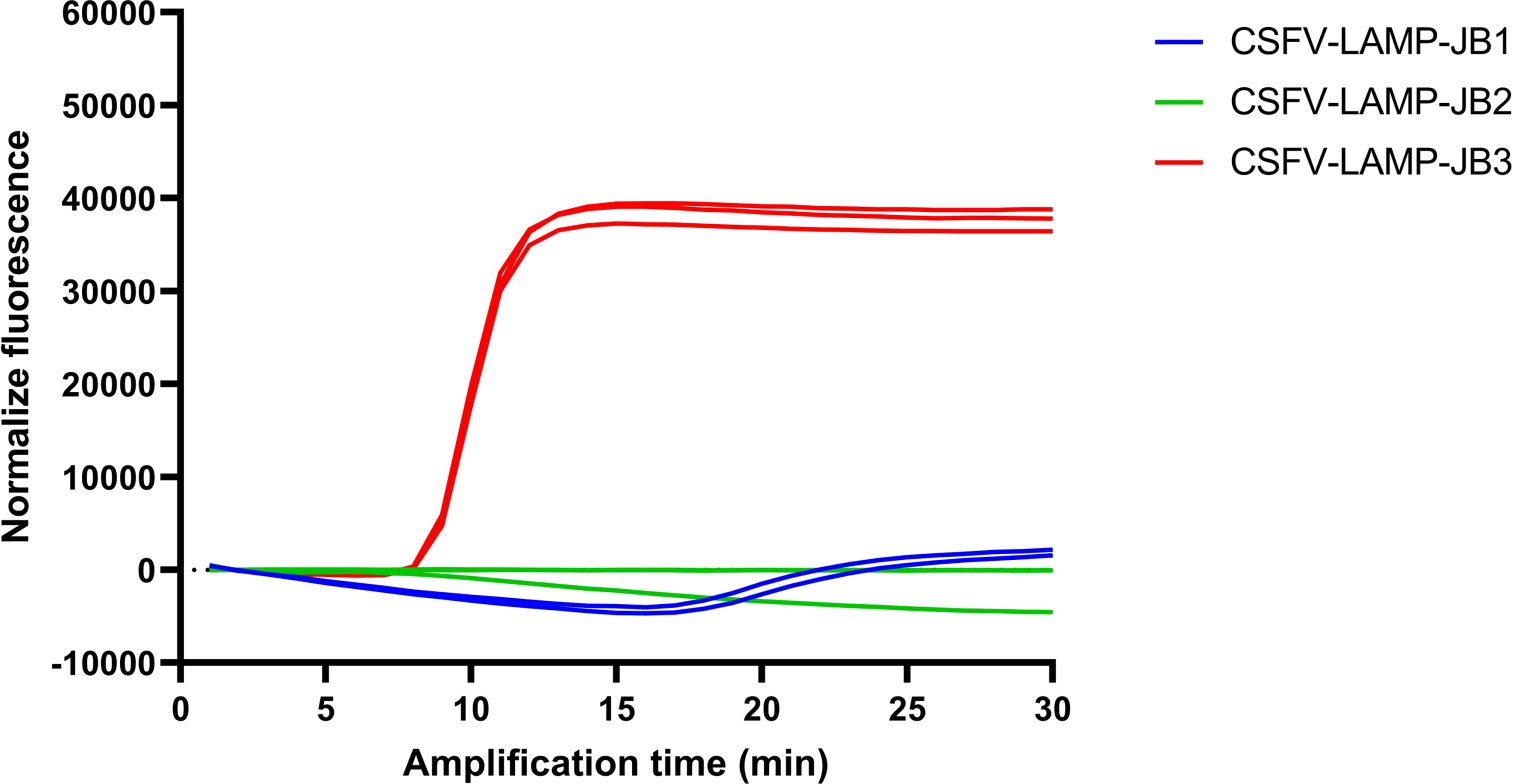
CSFV LAMP amplification by different primer sets. Fluorescent detection of LAMP amplification with the pSLC plasmid using the three different candidate primer sets. Amplification is expressed as normalized fluorescence vs the amplification time.

Primer sets JB1 and JB2 showed minor to no increase in fluorescence after 30 minutes (min) amplification, respectively, though a detectable increase in fluorescence was observed in the JB1 test between 15 and 20 min of amplification (**Figure 1**). Conversely, the CSFV-LAMP-JB3 primer set was shown to amplify the pSLC plasmid DNA with peak amplification detected at around 8 min. of amplification. Considering this, this primer set was selected for further validation.

### 3.2 The CSFV-LAMP-JB3 primer set shows stable plasmid amplification at multiple temperatures

**Figure 2:**
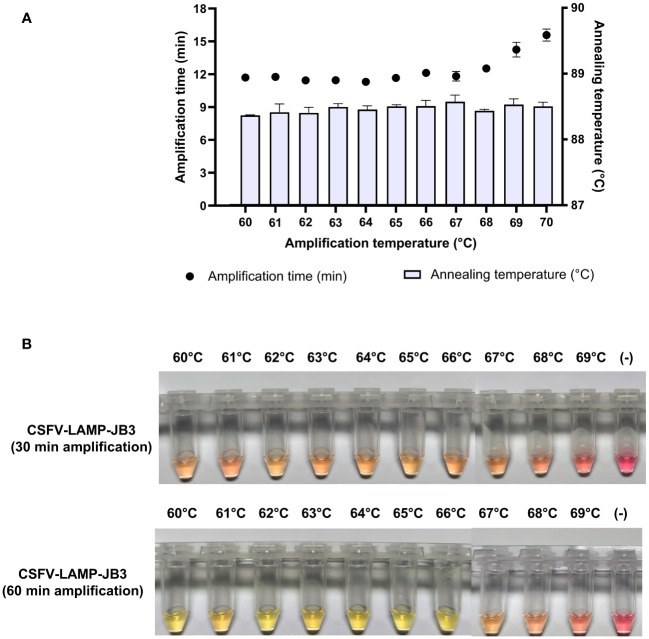
Thermal stability of CSFV-LAMP-JB3. **(A)** Fluorometric detection of CSFV-LAMP-JB3 at different temperatures, dots represent the time to detection (in minutes, left Y axis) and bars represent the annealing temperature for each reaction (in °C, right Y axis). **(B)** Colorimetric detection of CSFV-LAMP-JB3 at different temperatures. Reaction was stopped and documented at 30 (upper panel) and 60 (lower panel) minutes of amplification. Samples were deemed as positive when a discernible color change could be observed in the mastermix, compared with the negative control (far right tube).

The thermal stability of the candidate primer set was tested at a range between 60 and 70°C. Fluorometric amplification of the pSLC plasmid (10^4^ plasmid copies/ml) was detected at all the temperatures tested. Detection time was below 12 min for the majority of evaluated temperatures, but increased after 68°C. Annealing temperature was stable for all the samples (88.5 ± 0.2°C) (**Figure 2A**).

In the colorimetric detection, after 30 min amplification, detectable color changes were observed between 60 and 68°C, though none of the samples could be characterized as clearly positive (bright yellow coloration). Following an additional 30 min of amplification, samples amplified between 60 and 66°C were clearly positive. Samples amplified at higher temperatures were characterized as doubtful (pale pink coloration), while still being differentiated from the negative amplification control (**Figure 2B**).

### 3.3 The CSFV-LAMP reaction is able to detect plasmid DNA at low copy numbers and across various plasmid DNA-spiked matrices

**Figure 3:**
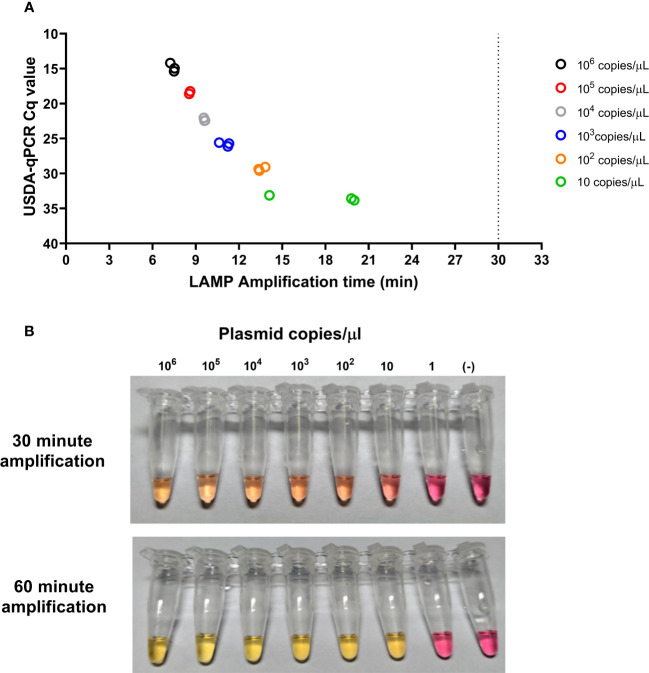
Analytical limit of detection for the CSFV-LAMP-JB3 assay. Ten-fold dilutions of the pSLC plasmid were tested by fluorometric **(A)** and colorimetric **(B)** LAMP, as well as by the USDA-reference RT-qPCR test. For the fluorometric detection, results are represented as the time to detection (in minutes, X axis), compared to the RT-qPCR results, expressed in Cq value (Y axis). Samples that showed LAMP amplification before 30 minutes (dotted line), were considered as positive. Colorimetric reactions were checked after 30 and 60 minutes of amplification and were deemed as positive when a discernible color change could be observed in the mastermix, compared with the negative control (far right tube).

When tested using different plasmid concentrations, the CSFV-LAMP-JB3 primer set proved consistently capable of detecting as low as 10 plasmid copies/μL, via fluorometric detection, corresponding with a Cq value of around 33 by the USDA-validated CSFV RT-qPCR assay (Eberling et al., 2011). In the colorimetric detection assay, noticeable color changes were observed in the master mix when the reaction was performed with as low as 10 plasmid copy/μL, after 30 to 60 min of amplification (**Figure 3**).

**Figure 4:**
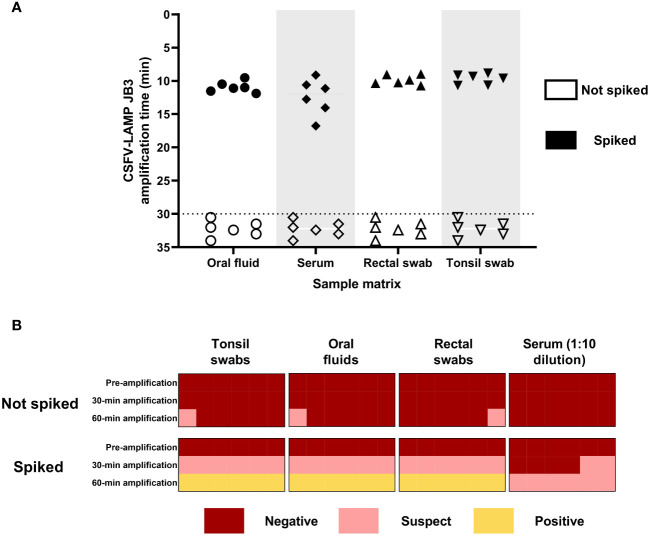
Detection of pSLC DNA in spiked matrices by CSFV-LAMP-JB3. **(A)** Fluorometric detection of pSLC DNA. Results are represented as time to amplification for each spiked (filled shapes) and not spiked (empty shapes) sample. **(B)** Colorimetric detection of ASFV-LAMP-BG3 amplification. Reactions were characterized as positive, suspect, or negative, according to the coloration of the mastermix after 30 or 60 min of amplification.

The CSFV-LAMP assay was then tested with sample matrices that are routinely used in surveillance, using the pSLC plasmid to spike them. The fluorometric detection method showed amplification for all the plasmid DNA-spiked samples, ranging from 8 to 20 minutes for amplification (**Figure 4A**). Meanwhile, the colorimetric test showed reliable detection of the spiked samples in three out of the four matrices tested, after 60 minutes of amplification. The remaining matrix (serum sample), while never reaching bright yellow coloration, was also clearly differentiated from the negative controls (**Figure 4B**).

### 3.4 The CSFV-LAMP assay can detect CSFV strains of different genotypes

**Figure 5:**
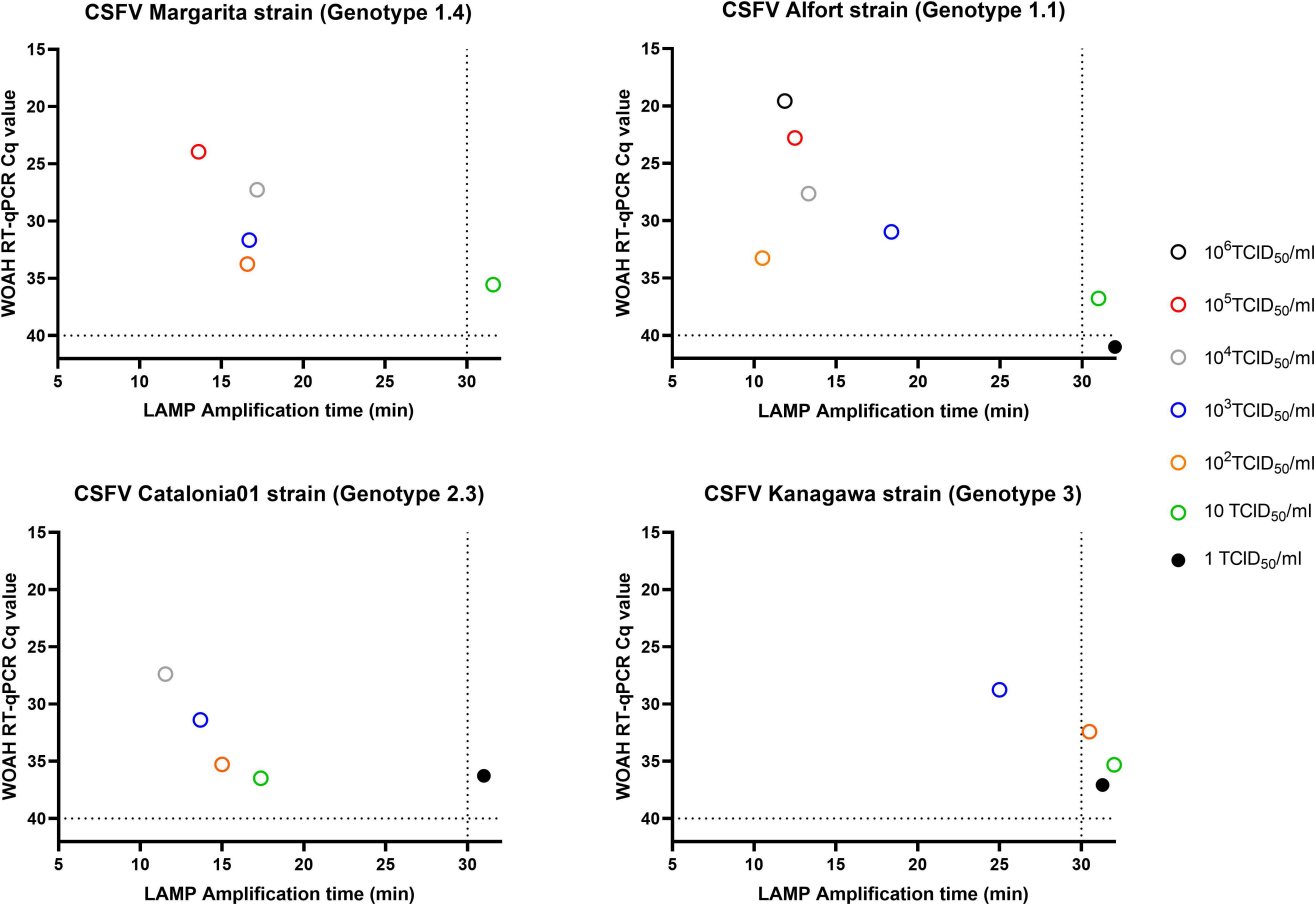
Detection of CSFV genotypes 1, 2 and 3 by fluorometric LAMP. Ten-fold dilutions of viral cultures from CSFV Margarita (genotype 1.4, upper left panel), Alfort-187 (genotype 1.1, upper right panel), Catalonia01 (genotype 2.3, lower left panel) and Kanagawa (genotype 3.4, lower right panel) strains were evaluated by the CSFV-LAMP-JB3 primer set. Results are represented as the time to detection in minutes (X axis) in comparison with the Cq value from the WOAH recommended qPCR assay (Y axis). Samples that showed LAMP amplification before 30 minutes (vertical dotted line), were considered as positive. Horizontal dotted line corresponds with limit of detection for the RT-qPCR.

Cell cultures infected with either of the three CSFV genotypes were tested using the CSFV-LAMP-JB3 primer set. In the fluorometric detection, genotype 1 and 2 strains tested were detected at concentrations as low as 100 and 10 TCID_50_/ml, respectively. This corresponded with Cq values between 33 and 36 by the RT-qPCR described by Hoffmann et al. (2005). Detection of the genotype 3 CSFV strain (Kanagawa) proved to have a higher detection limit, with all dilutions below 1000 TCID_50_/ml being negative (**Figure 5**).

Colorimetric LAMP showed improved detection compared to the fluorometric assay. In genotype 1 strains, the assay was able to detect samples at concentrations as low as 10^0,5^ TCID_50_/ml (Pinar del Rio strain), with the highest limit of detection (LOD) being 10^1,7^ TCID_50_/ml (Alfort-187). The LOD for these strains corresponded with Cq values between 32.95 and 36.77 (**Table 3**). In the case of genotype 2 strains, six out of the seven strains tested had a LOD on 1 TCID_50_/ml or lower, the only exception being Paderborn strain. In all these samples, the lowest concentration detected by colorimetric LAMP corresponded with the lowest concentration detected by RT-qPCR, with Cq values between 32.78 and 36.51 (**Table 3**). The CSFV genotype 3 strain tested, Kanagawa, was deemed as positive by colorimetric LAMP with a concentration as low as 10 TCID_50_/ml (Cq value of 35.3 by RT-qPCR).

**Table 3:** Colorimetric LAMP limit of detection.

Reference Strain	Genotype	LOD in colorimetric LAMP(TCID_50_/ml)	Cq Value by RT-qPCR	LOD in RT-qPCR (TCID_50_/ml)	Cq value by RT-qPCR
Alfort-187	1.1	10^1.7^	36,77	10^1.7^	36,77
Brescia	1.2	10^1.75^ – 10^0.75^	33,22 – 36,75	10^0.75^	36,75
Baker	1.2	10^1,4^	33,71	10^1,4^	33,71
Pinar del Rio	1.4	10^0.5^	36,33	10^0.5^	36,33
Margarita	1.4	10^1.3^	33,3	10^1.3^-10^0.3^	33,3 – 37,6
C-Strain	1.1	10	32,95	1	35,77
Catalonia 01	2.3	10^0,6^	34,17	10^0,6^	34,17
Diepholz	2.3	1	36,51	1	36,51
Italy	2.2	10^0.5^	35,32	10^0.5^	35,32
Paderborn	2.1	10^2^	33,285	10	35,35
Rostock	2.3	1	35,3	1	35,5
Uelzen	2.3	10^0,9^	34,24	10^0,9^	34,24
Spreda	2.3	10^0,7^	32,78	10^0,7^	32,78
Kanagawa	3.4	10	35,3	1	37,06

### 3.5 The CSFV-LAMP assay showed high exclusivity

The CSFV-LAMP assay was negative by both fluorometric and colorimetric detection for all non-Pestivirus pathogens tested, including ASFV. The technique was also unable to detect viral RNA from other pestiviruses affecting swine, such as BVDV-I, BVDV-II and BDV.

Non-specific LAMP detection was observed in serial dilutions of OVPV. In the fluorometric LAMP, an increase in normalized fluorescence accompanied by melting temperature detection (around 88.5°C) was evidenced in the dilutions corresponding with 10^4^-10^2^ TCID_50_. When tested by the CSFV RT-qPCR, these samples showed Cq values on 23.81, 27.74 and 31.31 for the 10^4^, 10^3^ and 10^2^ TCID_50_, respectively. Even though the increase in fluorescence was observed in these samples by fluorometric LAMP, they did not reach the level of fluorescence to be reported as positive by the Optigene software. In the colorimetric assay, a pale pink coloration was observed in the 10^4^ TCID_50_ dilution, corresponding with a doubtful result, being all other dilutions negative.

### 3.6 Detection of CSFV RNA in clinical samples from infected pigs by CSFV-LAMP

The fluorometric and colorimetric detection methods for the CSFV-LAMP-JB3 primer set were compared side by side with the CSFV RT-qPCR, using clinical samples from animals experimentally infected either genotype 1 or genotype 2 CSFV strains (Margarita and Cat01, respectively). None of the samples tested were positive before infection.

**Figure 6:**
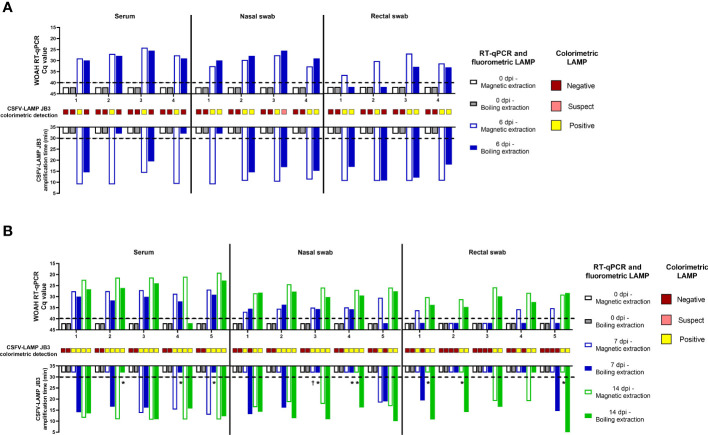
Clinical samples from CSFV experimental *in vivo* infection. Samples from pigs infected with the Margarita strain (genotype 1, **(A)** or with the Catalonia01 strain (genotype 2, **(B)** were tested by CSFV-LAMP-JB3, using fluorometric and colorimetric detection, as well as by the WOAH-recommended RT-qPCR. Fluorometric LAMP results are represented as time to amplification with each bar representing one sample (downward facing bars) at either the day of infection (grey scale bars), 7 dpi (blue scale bars) or 14 dpi (green scale bars). Each downward facing bar corresponds with an upward facing bar, representing the RT-qPCR result, expressed as Cq value. Dotted lines represent the limit of detection for each technique. Empty bars correspond with samples that had been magnetic-extracted, whereas filled bars represent boiling-extracted matrices. Squares in between the two sets of bars represent the colorimetric LAMP result for that sample. Colorimetric LAMP results are shown in a qualitative scale. Asterisk indicates samples that had clear amplification peaks but failed to be detected by the Optigene software, while showing melting temperature of 85.5 ± 0.5°C. Cross indicates samples with melting temperatures outside the range for positivity.

In the samples from animals infected with CSFV Margarita strain, the three viral RNA detection techniques were positive in all matrices analyzed through magnetic-based RNA extraction. When viral RNA was released through boiling samples (diluted at a 1/100 ratio in water) for 10 minutes, the WOAH CSFV RT-qPCR was successful in detecting viral RNA in serum and nasal swab samples but failed to detect two out of the four rectal swabs tested. Conversely, fluorometric LAMP detection was positive for all four rectal swab samples but was negative in one nasal swab and two serum samples. Colorimetric LAMP failed to detect any of the serum samples extracted through the boiling method, while proving effective in detection of all nasal swab samples and three out of four rectal swabs extracted through this method (**Figure 6A**).

In the Cat01-infected pigs, magnetic-extracted RNA from serum samples after infection, was positive for all animals by the CSFV RT-qPCR, while two of these samples were negative by fluorometric LAMP assay, both at 7 dpi (**Figure 6B**). When the same samples were subjected to boiling RNA release, after 1/100 dilution in sterile water, all of them were positive by RT-qPCR and fluorometric LAMP assay. All serum samples collected after infection were positive by colorimetric LAMP detection, regardless of extraction method. For the nasal swabs tested, RT-qPCR was positive in all but one of the animals (pig 5 at 7 dpi, boiling extraction), using either magnetic or boiling extraction. In the fluorometric LAMP all boiling-extracted RNA nasal swabs samples were positive at 7 dpi, while four extracted through magnetic extraction were negatives. At 14 dpi all nasal swab samples were positive by both extraction methods. Colorimetric LAMP assay was positive in all magnetic-extracted RNA both at 7 and 14 dpi, while 2 of the boiled samples were negative at 7 dpi (pig 1 and 5). In the case of rectal swabs, at 7 dpi, only two magnetic-extracted RNA, samples were negative by RT-qPCR, while all samples were negative by fluorometric LAMP. However, when boiled samples were evaluated, all rectal swabs were negative by RT-qPCR, and two samples were detected as positive by fluorometric LAMP. All rectal swabs collected at 14 dpi were positive by RT-qPCR and colorimetric LAMP, regardless of extraction method. Four out of these five samples were positive by fluorometric LAMP, using magnetic extraction, whereas all the five samples were positive, using boiling extraction.

### 3.7 Evaluation of the new LAMP assay in tissue samples from CSFV infected pigs

**Figure 7:**
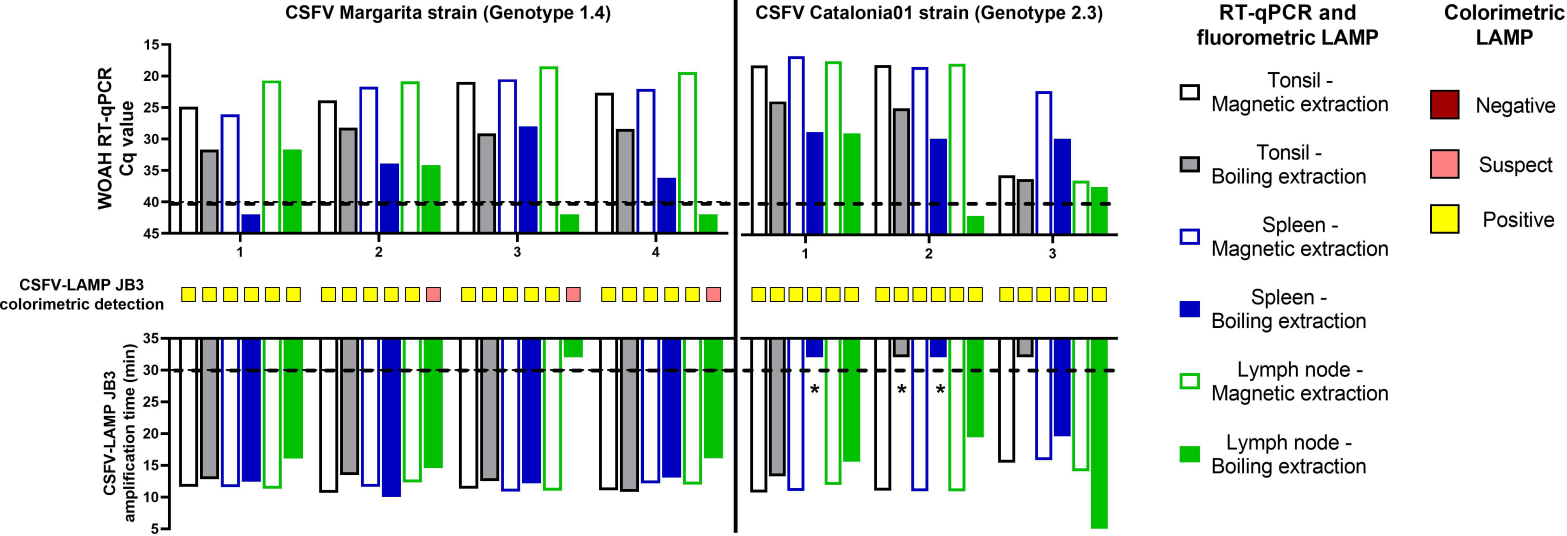
Tissue samples from CSFV experimental *in vivo* infection. Samples from pigs infected with the Margarita strain (genotype 1, left panel) or with the Catalonia01 strain (genotype 2, right panel) were tested by CSFV-LAMP-JB3, using fluorometric and colorimetric detection, as well as by the WOAH-recommended RT-qPCR. Fluorometric LAMP results (downward facing bars) are represented as time to amplification with each bar representing either tonsil (grey scale bars), spleen (blue scale bars) or mesenteric lymph node (green scale bars). Each downward facing bar corresponds with an upward facing bar, representing the RT-qPCR result, expressed as Cq value. Dotted lines represent the limit of detection for each technique. Empty bars correspond with samples that had been magnetic-extracted, whereas filled bars represent boiling-extracted matrices. Squares in between the two sets of bars represent the colorimetric LAMP result for that sample. Colorimetric LAMP results are shown in a qualitative scale. Asterisk indicates samples that had clear amplification peaks but failed to be detected by the Optigene software, while showing melting temperature of 85.5 ± 0.5°C.

Tonsil samples from Margarita infected pigs were positive by all three detection methods evaluated (RT-qPCR and fluorometric and colorimetric LAMP), regardless of the extraction method (**Figure 7**). Cq values for the RT-qPCR ranged between 20-25 in the magnetic-extracted samples and from 29 to 32 in the tissues subjected to boiling RNA release (1:100 dilution). In the case of fluorometric LAMP, amplification time was between 10-12 minutes and 10-14 minutes for the magnetic and boiled-extracted samples, respectively. In the spleen samples, all animals tested were positive by RT-qPCR in the magnetic-extracted RNA (Cq values between 20 and 27), however, one of them was negative when extracted by the boiling method. All spleen samples were positive by both fluorometric and colorimetric LAMP, in both extraction methods, with higher amplification times for the boiled-extracted RNA. Mesenteric lymph-nodes from infected animals were positive by all three detection methods, when using magnetic-extracted RNA. Meanwhile, in the boiling-extraction samples, two were negative by RT-qPCR and three were suspect by colorimetric LAMP, while all samples, but one boiled LN, were positive by the fluorometric assay.

For the samples from Cat01-infected pigs, the RT-qPCR detected viral RNA in all the tissue samples tested, using both RNA extraction methods, except for mesenteric lymph-node from pig 2, which was negative in the boiled-extracted RNA (**Figure 7**). Fluorometric LAMP detection was successful for all the magnetic-extracted RNA samples from these animals, while only one boiling-extracted sample (tonsil from pig 4) was negative. Colorimetric LAMP detection was positive for all tissue samples tested from pigs infected with the Cat01 CSFV strain.

## 4 Discussion

Rapid and accurate diagnosis remains the cornerstone of any disease control program in human or animal health (Wang et al., 2020a). This is particularly important in the case of diseases such as CSF, which are so impactful as to require mandatory notification to the WOAH (Mansour et al., 2015; World organisation for animal health (WOAH), 2019b). It is therefore highly important to improve the detection protocols, which can be achieved by enhancing either the diagnostic algorithms or techniques used (Wang et al., 2020a; Gabaldón-Figueira et al., 2023). LAMP poses an interesting addition to the array of diagnostic tools for diseases that require rapid detection, given its potential to be used as a point of care test (Chaouch, 2021; Moehling et al., 2021; Papadakis et al., 2022; Atceken et al., 2023). This type of detection would greatly reduce the time to obtain results, allowing for faster containment of affected farms. The present work shows the development of a diagnostic technique that may have an impact on these two aspects. The new CSFV LAMP assay (using the CSFV-LAMP-JB3 primers set) proved capable of reliably detecting RNA from multiple CSFV genotypes, even at low concentrations, and using different RNA extraction methods.

The challenges of developing a nucleic acid based CSFV diagnostic technique are many, considering that this is an RNA virus, and its genetic material is, therefore, more unstable and prone to mutation than other pathogens. Moreover, the genome size is relatively small (approximately 12.6 kb), so finding a large uninterrupted conserved genomic region, or multiple adjacent smaller conserved regions, that allow for the generation of LAMP primers can be difficult. This is reflected in the fact that the primer designer software suggested sequences that were highly repetitive and overall unsuccessful in amplifying the pSLC DNA or the viral RNA. In addition, similar sequences to some of the primers used in the present study were found to have been previously reported for CSFV-LAMP applications, specifically the F3 (Chowdry et al., 2014) and B1c-B2 (Wongsawat, 2010) regions from JB3. The remaining sequences from the CSFV-LAMP-JB3 have not been previously reported for this application, to the best of our knowledge at the time of writing.

Due to the extensive collection of CSFV strains and expertise at the WOAH Reference Laboratory for CSF in IRTA, efforts on this assay development were divided between the two institutions in the present work. UIUC optimized primer design and performed initial testing, based on the pSLC plasmid, and using the diagnostic technique for comparison that is routinely used in this institution for diagnostic surveillance (Eberling et al., 2011). Meanwhile, testing and optimization of the LAMP assay with various live CSFV strains and infected tissues requires biosafety level 3 (BSL3) containment and thus was performed at IRTA, using the RT-qPCR for validation (Hoffmann et al., 2005), which has been certified under ISO 17025, a high-quality standard. The fact that the LAMP assay has been successfully compared with two different RT-qPCR tests used for surveillance in multicontinental settings, adds to the robustness of the work.

The detection of multiple CSFV genotypes by the CSFV-LAMP assay is highly encouraging in efforts to develop such a diagnostic test. In this regard, it is noteworthy that detection of different genotypes, while being successful, was not the same, since the fluorometric LAMP assay proved to have a higher limit of detection for genotype 3 (10^3^ TCID_50_/ml) than for the other two genotypes tested. Interestingly, colorimetric LAMP detection proved to be of similar analytical sensitivity to that reported by one of the WOAH-recommended CSFV RT-qPCR tests (Hoffmann et al., 2005). In addition, detection of viral RNA appeared to be much faster, in the colorimetric assay, than detection of the pSLC plasmid. This might be related to a higher homology of the primers with the field strain RNA than the plasmid sequence, given that the plasmid was not designed to be homologous to any individual strain, but rather to have as close as a consensus sequence as possible for that specific genomic region. Differences in sensitivity of the fluorometric and colorimetric detection may relate to the efficiency of the reverse transcriptase polymerases as well as the pH sensitivity of the colorimetric method.

There have been previous attempts at developing a CSFV LAMP assay, dating as far back as 2009 (Chen et al., 2009; Yin et al., 2010; Wongsawat et al., 2011; Chowdry et al., 2014; Postel et al., 2015; Tran et al., 2023). However, the feasibility to apply some of these techniques in the field has been mostly limited by the use of extracted RNA, which implies training and infrastructure that are not readily available in field conditions. Additionally, some of these techniques lack reliable detection across multiple genotypes, or a detection method that can be employed in the field. Conversely, the CSFV-LAMP assay from the present study can be performed using minimal equipment. Fluorometric detection can be achieved via an easily portable machine, whereas colorimetric detection does not require any detection equipment whatsoever, with a heating source, such as a warm bath, being the only requirement. Recently, a new LAMP technique using body warmers as an alternative to perform colorimetric LAMP reactions without the use of electricity has been proposed to detect CSFV (Tran et al., 2023). Considering that the CSFV-LAMP test developed here proved to be efficient at a wide range of temperatures, similar to those reported for the body warmers, this would be an easily adaptable feature to further improve the technique proposed in the present study. The fact that the primer design for CSFV-LAMP-JB3 is based on the same target sequence as the RT-qPCR techniques previously validated for the CSFV official molecular diagnosis (Hoffmann et al., 2005; Eberling et al., 2011), provides further evidence of its reliability.

The results from samples belonging to experimentally infected pigs reveal, at once, the advantages and possible limitations of the CSFV-LAMP assay. On the one hand, it’s worth highlighting that the LAMP assay, whether by fluorometric or colorimetric detection, showed the similar sensitivity as the CSFV RT-qPCR assay reported by (Hoffmann et al., 2005), when evaluating magnetic extracted RNA samples from genotype 1. Conversely, the use of this CSFV-LAMP test in the field using minimal equipment, particularly by colorimetric detection, may be limited when trying to analyze serum samples, since all of these were negative when evaluating boiled serum samples. This may be due to the buffering capabilities of serum, which hinder the variation in the mastermix pH and, therefore, the colorimetric change. Similar issues in colorimetric LAMP assays have been reported in a previous study (Bohorquez et al., 2023). This phenomenon can usually be solved by serum dilution, as was the case with the samples from animals infected with CSFV genotype 2 Catalonia strain. Nevertheless, this potential limitation can be overcome by the use of other minimally invasive samples, such as nasal and rectal swabs, that did not show these problems and can be collected in a less invasive manner and with very little training in a farm setting. Additionally, the results using samples from pigs experimentally infected with the CSFV genotype 2 strain evidenced a better performance by the colorimetric detection method than the fluorometric. The colorimetric LAMP reaction coincided with 93.3% of the RT-qPCR results and was able to detect viral RNA in all samples, regardless of extraction method, after 14 days of infection. This is particularly encouraging, taking into account that the viral RNA load in some of the nasal and rectal swabs at 7 dpi was near the limit of detection of RT-qPCR.

A potential drawback of the CSFV-LAMP test lies on the non-specific detection of the new OVPV viral RNA. This result is not entirely surprising, given the close genomic proximity of OVPV with CSFV (Sozzi et al., 2019), as well as the fact that RNA from this virus can be detected by the CSFV RT-qPCR previously described by Hoffmann et al. (2005) (Wang et al., 2020b). However, the colorimetric results show that even a large amount of circulating viral load generates only a doubtful result. It is worth highlighting the capacity of this virus to rapidly generate antibodies in swine after infection (Wang et al., 2020b), which in any case would favor reducing the circulating viral load and in turn reducing the possibility of OVPV cross-reactivity with test developed here. In addition, it’s unlikely that the cross-reaction of the LAMP test with OVPV would translate to a field setting, since the only study regarding experimental infection of pigs with OVPV showed that the viral RNA load detected in pigs after infection is much lower than that used for testing of the LAMP assay in the present study (Bohórquez et al., 2021).

The results from the present study show the development of a novel tool for the detection of CSFV, one that may have important applications in a field setting, where subclinical and chronic forms of disease continue to persist (Coronado et al., 2019; Ganges et al., 2020). Some of these forms of disease fail to be diagnosed by the serological techniques routinely used for surveillance programs and thus their detection relies on nucleic acid amplification (Muñoz-González et al., 2015; Coronado et al., 2019). Further studies will be needed to continue the validation of the CSFV-LAMP, testing its accuracy in different matrices, as well as evaluating its performance in samples from the field. Similarly, validation by independent laboratories would be needed to consider the feasibility of widespread usage for such a technique, whether by itself or in combination with commonly used surveillance tests.

The combined use of novel and well-stablished diagnostic techniques, such as those used recommended for surveillance by government organizations, could prove to be a useful alternative for the early detection of CSFV, facilitating faster response time and leading to an overall positive impact in the eradication of the disease. Thus, official regulations and guidelines for the use of point-of-care diagnostic tests in surveillance and control programs for notifiable diseases for the WOAH need to be established. Future testing will also include stability of the kit and reagents under temperature extremes encountered in the field.

## Data availability statement

The original contributions presented in the study are included in the article/**Supplementary Material**. Further inquiries can be directed to the corresponding author.

## Ethics statement

The animal study was approved by the Ethics Committee for Animal Experiments of the Autonomous University of Barcelona (UAB) under number 8642, and the Ethical Committee of the Generalitat de Catalonia, Spain under project number 10908, according to existing national and European regulations. The study was conducted in accordance with the local legislation and institutional requirements.

## Author contributions

JB: Visualization, Investigation, Writing – review & editing, Writing – original draft, Validation, Methodology, Formal analysis, Data curation. AM-A: Writing – review & editing, Writing – original draft, Validation, Methodology, Investigation, Formal analysis, Data curation. SL: Writing – review & editing, Writing – original draft, Validation, Methodology. LC: Writing – review & editing, Writing – original draft, Validation, Methodology, Formal analysis, Data curation. RR: Writing – review & editing, Validation, Methodology. MA: Writing – review & editing, Validation, Methodology, Data curation. CM: Writing – review & editing, Writing – original draft, Validation, Supervision, Resources, Project administration, Methodology, Investigation, Funding acquisition, Formal analysis, Data curation. LG: Writing – review & editing, Writing – original draft, Validation, Supervision, Resources, Project administration, Methodology, Funding acquisition, Formal analysis, Data curation, Conceptualization.
